# Telephone versus in-person administration of the Early Childhood Development Index 2030: a crossover study, Brazil, Canada, India and Malawi

**DOI:** 10.2471/BLT.25.294390

**Published:** 2026-05-27

**Authors:** Nicholas Kuzik, Sabrina Buchanan, Sarita de Mendonça Bacciotti, Madison Boyd, Emily Bremer, Brianne A Bruijns, Hilary AT Caldwell, Claudia Cappa, Valerie Carson, Guy Faulkner, Alex Antonio Florindo, Anelise Reis Gaya, Kianoush Harandian, Uddhavi Kand, Janine Kayange, Himangi Lubree, Clarice Martins, Tawonga Mwase-Vuma, Anthony Okely, Linda S Pagani, Nicole Petrowski, Morgan Potter, Evelyn Helena Corgosinho Ribeiro, Patricia Tucker, Roseanne Autran, Mark S Tremblay

**Affiliations:** a Healthy Active Living and Obesity Research Group, Children’s Hospital of Eastern Ontario Research Institute, 401 Smyth Road, Ottawa, Ontario, K1H 8L1, Canada.; b Faculty of Education, Federal University of Mato Grosso do Sul, Campo Grande, Brazil.; c Faculty of Kinesiology, Sport, and Recreation, University of Alberta, Edmonton, Canada.; d School of Kinesiology, Acadia University, Wolfville, Canada.; e School of Occupational Therapy, Western University, London, Canada.; f Healthy Populations Institute, Dalhousie University, Halifax, Canada.; g Data and Analytics Section, UNICEF Office of Strategy and Evidence – Innocenti, Florence, Italy.; h School of Kinesiology, University of British Columbia, Vancouver, Canada.; i School of Arts, Sciences and Humanities, University of São Paulo, São Paulo, Brazil.; j Superior School of Physical Education and Physiotherapy, University of Pelotas, Pelotas, Brazil.; k School of Psycho-Education, University of Montreal, Montreal, Canada.; l Vadu Rural Health Program, KEM Hospital Research Centre, Pune, India.; m Centre for Social Research, University of Malawi, Zomba, Malawi.; n Research Centre of Physical Activity, Health and Leisure, University of Porto, Porto, Portugal.; o Early Start Institute, University of Wollongong, Wollongong, Australia.; p Physical Activity Epidemiology Group, University of São Paulo, São Paulo, Brazil.; q Faculty of Physical Education and Physiotherapy, Federal University of Amazonas, Manaus, Brazil.

## Abstract

**Objective:**

To examine agreement between telephone and in-person administration of the Early Childhood Development Index 2030 (ECDI2030) survey.

**Methods:**

In this crossover study, we compared in-person ECDI2030 interviews with other modalities (primarily telephone-based interviews) selected at a country level in Brazil, Canada, India and Malawi. Eligible participants were primary caregivers of preschool-aged children (36–60 months old), with equal recruitment across child sex and urban and rural residence. The primary outcome was agreement in overall scores between in-person interviews and other tested modalities, assessed using paired *t*-tests and intraclass correlation coefficients.

**Findings:**

The analytical sample included 258 individuals, with 212 paired observations for in-person and telephone-based interviews. Pooled *t*-tests showed no significant differences in overall scores between the interview modalities. However, in-person interviews yielded slightly higher scores than telephone interviews for female children (mean difference: 0.23; Cohen’s *d*_diff_: 0.22) and participants from India (mean difference: 0.47; Cohen’s *d*_diff_: 0.44). Canadian participants had lower scores on the second assessment completed (mean difference: −0.27; Cohen’s *d*_diff_: −0.36), regardless of modality. However, pooled and stratified intraclass correlation coefficients analyses indicated that these modalities were statistically similar, with values ranging from 0.89 to 0.93, which are values commonly categorized as good to excellent.

**Conclusion:**

This multinational, multicentre, crossover study shows high agreement between telephone and in-person administration of the ECDI2030 across economically, culturally and geographically diverse settings. Expanding administration modalities may improve feasibility, particularly in geographically dispersed populations and rural settings.

## Résumé

**Objectif:**

Examiner la concordance entre des entretiens par téléphone et en personne dans le cadre de l’enquête sur l’Indice de développement de la petite enfance 2030 (ECDI2030).

**Méthodes:**

Cette étude croisée a comparé les entretiens en personne dans le cadre de l’ECDI2030 à d’autres modalités (principalement des entretiens téléphoniques) sélectionnées au niveau national au Brésil, au Canada, en Inde et au Malawi. Les participants éligibles étaient les principaux gardiens d’enfants d’âge préscolaire (âgés de 36 à 60 mois). Le recrutement de ces participants était équilibré selon le sexe de l’enfant et le lieu de résidence (urbain ou rural). Le critère de jugement principal était la concordance des scores globaux entre les entretiens en personne et les autres modalités d’entretien, évaluées à l’aide de tests *t* appariés et de coefficients de corrélation intraclasse.

**Résultats:**

L’échantillon d’analyse comprenait 258 personnes, avec 212 observations appariées pour les entretiens en personne et les entretiens téléphoniques. Les tests *t* regroupés n’ont montré aucune différence significative dans les scores globaux entre les différentes modalités d’entretien. Cependant, les entretiens en personne ont généré des scores légèrement supérieurs à ceux des entretiens téléphoniques pour les enfants de sexe féminin (différence moyenne: 0,23; *d*_diff_ de Cohen: 0,22) et les participants indiens (différence moyenne: 0,47; *d*_diff_ de Cohen: 0,44). Les participants canadiens ont obtenu des scores plus faibles lors de la deuxième évaluation (différence moyenne: -0,27; *d*_diff_ de Cohen: −0,36), quelle que soit la modalité d’entretien. Toutefois, les analyses des coefficients de corrélation intraclasse regroupés et stratifiés ont indiqué que ces modalités étaient statistiquement similaires, avec des valeurs comprises entre 0,89 et 0,93, qui sont généralement évaluées comme bonnes à excellentes.

**Conclusion:**

Cette étude multinationale, multicentrique et croisée présente une forte concordance entre les entretiens téléphoniques et en personne de l’ECDI2030 dans des contextes diversifiés sur les plans économique, culturel et géographique. L’élargissement des modalités d’entretien pourrait améliorer la faisabilité, en particulier dans les populations géographiquement dispersées et les milieux ruraux.

## Resumen

**Objetivo:**

Examinar la concordancia entre entrevistas telefónicas y presenciales en el marco de la encuesta del Índice de desarrollo de la primera infancia 2030 (ECDI2030).

**Métodos:**

En este estudio cruzado, se compararon entrevistas presenciales del ECDI2030 con otras modalidades (principalmente entrevistas telefónicas) seleccionadas a nivel nacional en Brasil, Canadá, India y Malawi. Los participantes elegibles fueron cuidadores principales de niños en edad preescolar (de 36 a 60 meses de edad), con un reclutamiento equilibrado según el sexo del niño y el lugar de residencia (urbano o rural). El resultado principal fue la concordancia de las puntuaciones globales entre las entrevistas presenciales y las otras modalidades de entrevista evaluadas, analizada mediante pruebas *t* pareadas y coeficientes de correlación intraclase.

**Resultados:**

La muestra analítica incluyó 258 personas, con 212 observaciones pareadas correspondientes a entrevistas presenciales y telefónicas. Las pruebas *t* agrupadas no mostraron diferencias significativas en las puntuaciones globales entre las distintas modalidades de entrevista. Sin embargo, las entrevistas presenciales generaron puntuaciones ligeramente superiores a las entrevistas telefónicas en niñas (diferencia media: 0,23; *d*_diff_ de Cohen: 0,22) y en participantes de India (diferencia media: 0,47; *d*_diff_ de Cohen: 0,44). Los participantes de Canadá obtuvieron puntuaciones más bajas en la segunda evaluación realizada (diferencia media: -0,27; *d*_diff_ de Cohen: -0,36), independientemente de la modalidad de entrevista. No obstante, los análisis agrupados y estratificados de los coeficientes de correlación intraclase indicaron que estas modalidades eran estadísticamente similares, con valores comprendidos entre 0,89 y 0,93, generalmente considerados entre buenos y excelentes.

**Conclusión:**

Este estudio multinacional, multicéntrico y cruzado muestra una elevada concordancia entre las entrevistas telefónicas y presenciales del ECDI2030 en contextos diversos desde el punto de vista económico, cultural y geográfico. La ampliación de las modalidades de entrevista podría mejorar la viabilidad, especialmente en poblaciones geográficamente dispersas y en entornos rurales.

## ملخص

**الغرض:**

دراسة التوافق بين الإدارة عبر الهاتف والإدارة الشخصية لاستبيان مؤشر تنمية الطفولة المبكرة 2030 (ECDI2030).

**الطريقة:**

في هذه الدراسة المقارنة، قمنا بمقارنة المقابلات الشخصية لاستبيان ECDI2030 مع طرق أخرى (المقابلات الهاتفية بشكل أساسي)، تم تحديدها على مستوى كل دولة في البرازيل، وكندا، والهند، وملاوي. كان المشاركون المؤهلون هم مقدمي الرعاية الأساسيين لأطفال في سن ما قبل المدرسة (36 إلى 60 شهرًا)، مع مراعاة التوزيع المتساوي بين الجنسين ومكان الإقامة الحضري أو الريفي. كانت النتيجة الرئيسية هي التوافق في الدرجات الإجمالية بين المقابلات الشخصية والطرق الأخرى قيد الاختبار، وتم تقييمها باستخدام اختبارات *t *-tests المقترنة ومعاملات الارتباط داخل الفئة.

**النتائج:**

شملت العينة التحليلية 258 فردًا، مع 212 ملاحظة مقترنة للمقابلات الشخصية وعبر الهاتف. أظهرت اختبارات *t *-tests المجمعة عدم وجود فروق ملموسة في الدرجات الإجمالية بين أساليب المقابلة. مع ذلك، حققت المقابلات الشخصية درجات أعلى قليلًا من المقابلات عبر الهاتف بالنسبة للأطفال الإناث (متوسط الفرق: 0.23؛ معامل كوهين: 0.22) والمشاركين من الهند (متوسط الفرق: 0.47؛ معامل كوهين: 0.44). وسجل المشاركون الكنديون درجات أقل في التقييم الثاني (متوسط الفرق: -0.27؛ معامل كوهين: -0.36)، بغض النظر عن أسلوب المقابلة. ومع ذلك، أشارت تحليلات معاملات الارتباط داخل الفئة المجمعة والمصنفة إلى تشابه إحصائي بين هذه الأساليب، حيث تراوحت القيم بين 0.89 و0.93، وهي قيم تُصنف عادةً على أنها جيدة إلى ممتازة.

**الاستنتاج:**

إن هذه الدراسة المقارنة متعددة الجنسيات ومتعددة المراكز، تُظهر توافقًا عاليًا بين إدارة ECDI2030 عبر الهاتف وعبر المقابلات الشخصية في أوضاع متنوعة اقتصاديًا وثقافيًا وجغرافيًا. قد يؤدي توسيع أساليب الإدارة إلى تحسين الجدوى، لا سيما في المناطق ذات الكثافة السكانية المتفرقة جغرافياً والأوضاع الريفية.

## 摘要

**目的:**

旨在检验电话与面对面访谈这两种《儿童早期发展指数 2030》(ECDI2030) 调查实施方式的一致性。

**方法:**

在这项交叉研究中，我们将在巴西、印度、加拿大和马拉维实施的 ECDI2030 面对面访谈与在相应国家层面选择的其他访谈方式（主要是电话访谈）进行了比较。入选参与者是学龄前儿童（36-60 个月大）的主要照护者，同时基于儿童性别和城乡居住地进行了均衡招募。主要结局指标是面对面访谈与其他受试方式在总分方面的一致性，该一致性使用配对 *t* 检验和组内相关系数进行评估。

**结果:**

分析样本包含 258 人，其中有 212 人被纳入了面对面访谈和电话访谈的配对观察。合并* t *检验结果显示，两种访谈方式的总分并无明显差异。但是，针对女童（均数差：0.23；Cohen’s *d_diff_*：0.22）和印度参与者（均数差：0.47；Cohen’s *d_diff_*：0.44）的面对面访谈得分略高于电话访谈。在第二次评估中，加拿大参与者在所有访谈方式下的得分均较低（均数差：−0.27；Cohen’s *d_diff_*：−0.36）。但是，合并及分层组内相关系数分析表明，这些访谈方式在统计学上具有相似性，其值介于 0.89 至 0.93 之间，而处于这个范围内的值通常被归入“良好至优秀”一类。

**结论:**

这项跨国多中心交叉研究表明，在经济、文化和地理环境迥异的条件下实施的 ECDI2030 面对面访谈和电话访谈存在高度一致性。拓展调查实施方式可提高可行性，特别是在人口分布比较分散的地区和农村地区。

## Резюме

**Цель:**

Исследовать согласованность результатов оценки популяции по показателю развития детей раннего возраста 2030 (ECDI2030), проводимой в телефонном режиме и при личном контакте.

**Методы:**

В этом перекрестном исследовании авторы сравнили проводимые лично анкетирования по ECDI2030 с опросами в других модальностях (в основном в телефонном режиме), выбранными на уровне таких стран, как Бразилия, Индия, Канада и Малави. В опросах участвовали в основном лица, осуществляющие уход за детьми дошкольного возраста (от 36 до 60 месяцев) с равным представительством в отношении пола детей и проживания их в сельской или городской местности. Основной результат показал, что общий балл анкеты является сопоставимым при личном анкетировании и проведении опроса в других модальностях; для оценки использовался парный t-критерий и коэффициенты внутриклассовой корреляции.

**Результаты:**

Аналитическая выборка включала 258 лиц, при этом было проведено 212 парных наблюдений процесса анкетирования, который проводился как при личном контакте, так и в телефонном режиме. Объединенные *t*-критерии не показали значимой разницы в общих баллах анкетирования для разных модальностей. Однако при личном анкетировании были получены несколько более высокие баллы по сравнению со случаями, когда опрос проводился в телефонном режиме, если речь шла о девочках (средняя разница: 0,23; коэффициент Коэна *d*_diff_: 0,22), а также в тех случаях, когда анкетируемые были из Индии (средняя разница: 0,47; коэффициент Коэна *d*_diff_: 0,44). Участники из Канады имели более низкий результат по второму проведенному оцениванию (средняя разница: –0,27; коэффициент Коэна *d*_diff_: –0,36), независимо от модальности. Однако анализ объединенных и стратифицированных коэффициентов внутриклассовой корреляции показал, что эти модальности статистически сопоставимы (значения варьировались в пределах от 0,89 до 0,93, что обычно означает степень корреляции от хорошей до отличной).

**Выводы:**

Это многонациональное, многоцентровое перекрестное исследование показало высокую степень согласованности результатов при проведении анкетирования по ECDI2030 методом личного контакта и в телефонном режиме в странах с разными экономическими, культурными и географическими условиями. Расширение используемых модальностей анкетирования может улучшить его практическую осуществимость, в частности в географически разбросанных популяциях и сельской местности.

## Introduction

Early childhood development is foundational in shaping academic achievement, economic prosperity, public health and humanitarian progress; it affects individuals, communities, nations and the world at large.^1^^–^^3^ This sensitive period of human development influences lifelong outcomes across multiple domains, for example social, emotional, physical domains.^4^ Consequently, the importance of early childhood development was recognized in Target 4.2 of the United Nations sustainable development goals.^5^ Target 4.2 aims to ensure that, by 2030, every girl and boy has access to quality early childhood development, care and pre-primary education to support readiness for primary education.^5^ Within this target, indicator 4.2.1 focuses on developmental readiness by measuring the proportion of children aged 24–59 months who are on track in health, learning and psychosocial well-being, disaggregated by sex, using the Early Childhood Development Index 2030 (ECDI2030). This index, developed by the United Nations Children’s Fund (UNICEF), the custodian agency for indicator 4.2.1, is a simple, comprehensive population-level questionnaire designed to estimate overall development among children aged 24–59 months across the three interrelated domains of health, learning and psychosocial well-being.^6^

Beyond tracking progress towards the sustainable development goals, a holistic and brief assessment of childhood development could be a very promising tool for researchers worldwide. In 2020, UNICEF developed, tested and validated the ECDI2030 for implementation as an in-person interview.^6^ However, limiting its administration to in-person interviews could pose challenges for participant recruitment and retention, data completeness and overall feasibility. Since the coronavirus disease 2019 pandemic, interest in alternatives to in-person assessments has grown.^7^ Thus, testing alternative modalities for administering the ECDI2030 questionnaire could support researchers seeking to assess the foundations of learning, health and psychosocial well-being.

As it cannot be assumed that psychometric properties will inherently transfer across administration methods, different methods should be examined.^8^ A recent study in Nepal compared the concurrent validity of administering the ECDI2030 as a telephone or in-person interview.^9^ The study included 364 primary caregivers of children aged 24–59 months and randomized them to either in-person or telephone administration of the ECDI2030. The study found that overall scores, the percentage of children classified as developmentally on track and individual question scores were equivalent across telephone-based and in-person interviews. Thus, the researchers concluded that the ECDI2030 could be administered by telephone or in person in Nepal. While the results are promising, it remains unknown whether the findings can be generalized beyond Nepal or extended to other administration modalities, such as virtual video interviews, paper questionnaires or online questionnaires.

Perhaps one of the greatest strengths of the ECDI2030 is that UNICEF developed and validated it using international samples, as opposed to previous measures validated in predominantly Western, urban and Caucasian populations.^10^ To date, the ECDI2030 is readily accessible and validated in eight languages: Arabic, Chinese, English, French, European and Brazilian Portuguese, Russian and Spanish.^6^ The SUNRISE study^11^ provides an assessment platform to extend the validation testing of the ECDI2030 as it also emphasizes global generalizability; it includes 68 countries and assesses global adherence to World Health Organization (WHO) guidelines on physical activity, sedentary behaviour and sleep for children younger than 5 years.^12^ Additionally, the SUNRISE study uses comprehensive, well-established assessments of motor and cognitive skills as indicators of development, and ensures that each country’s sample equally represents rural and urban girls and boys aged 3–4 years.^11^

Countries participating in SUNRISE that use languages validated by ECDI2030 have the opportunity to incorporate ECDI2030 into the study protocol. Doing so would allow future comparisons between overall ECDI2030 development scores and the more comprehensive developmental measures already included in SUNRISE. However, incorporating the ECDI2030 into the existing SUNRISE data collection protocol may not be feasible if in-person interviews are the only available administration method. To improve feasibility and participant response rates, the SUNRISE study uses flexible questionnaire administration methods, such as telephone interviews and online questionnaires. This flexibility is particularly important in rural areas distant from research centres and in settings where low literacy and poor internet connectivity pose logistical challenges. Examining different ECDI2030 administration modalities is an important first step in assessing whether this survey can be incorporated into the existing SUNRISE data collection protocol. Accordingly, we aimed to assess the concurrent validity of different ECDI2030 administration modalities.

## Methods

### Study design

This multicentre, multinational, crossover study compared in-person ECDI2030 interviews with telephone interviews and online questionnaires. We selected a crossover design to enable within-participant comparisons, thereby eliminating variability between participants. We used a 1-week washout period between assessments to limit carryover (memory) effects while avoiding a delay long enough for child development to progress to the point where scores could change.

Eligible participants were primary caregivers of preschool-aged children aged 36–60 months. The specification of mother or primary caregiver is in line with UNICEF’s guidance for implementing the ECDI2030,^6^ while the age requirement aligns with the target sample of the SUNRISE study. The study included four countries: Brazil, Canada, India and Malawi. Together, these countries represent all World Bank income classifications (high-income: Canada; upper-middle income: Brazil; lower-middle income: India; and low-income: Malawi), different continents and both hemispheres (Northern: Canada and India; Southern: Brazil and Malawi). All participating caregivers provided written informed consent and each study site obtained ethical approval.

UNICEF trained country leads and key data collectors on ECDI2030 administration; the study team (coauthors) then drafted a proposal to examine various modalities for administering ECDI2030 in Canada. Once the Canadian team leaders had finalized the proposal, the other country leads adapted the protocol to reflect the nuances of data collection in their respective countries (Table 1). The original proposal included five modalities of ECDI2030 administration: (i) telephone interviews; (ii) virtual video interviews using Zoom (Zoom Communications, Inc., San Jose, United States of America) or Microsoft Teams (Microsoft, Redmond, USA); (iii) paper-based questionnaires; (iv) online questionnaires completed via REDCap;^17^ and (v) in-person interviews. We treated in-person interview as the criterion measure, because UNICEF originally developed ECDI2030 for in-person administration;^6^ the primary outcome was the difference in overall development scores between the criterion measure and the other tested modalities. Additionally, we grouped the modalities as either interview-based (telephone, in-person and virtual video) or self-directed (paper-based and online questionnaires). We also developed a brief questionnaire to collect basic demographic information on the primary caregiver and child, such as child ethnicity, sex and age. Finally, we included issues previously described in Nepal to compare administration difficulties associated with the different interview modalities: (i) difficulties to assure that the interview took place in a private and quiet place to avoid others’ interference; (ii) any interruptions or disruptions of the interview; (iii) difficulties to assure the respondent answered the questions solely based on their perception and knowledge of the child, without directly checking with the child or consulting with other household members; and (iv) difficulties to check whether respondents seemed to misinterpret or not understand the questions.^9^

**Table 1:** Protocol and order assignment for comparing Early Childhood Development Index 2030 modalities, Brazil, Canada, India, Malawi, June 2023–February 2025

Country, recruitment and study location	Sample and recruitment period	Urban and rural definition	Modalities tested	Order of testing modalities
**Brazil**				
Sites represented the Brazilian Institute of Geography and Statistics (IBGE) surveillance regions: South-east, North-east, South, North and Mid-west. Site locations included Salesópolis (South-east), João Pessoa (North-east), Canoas and Canguçu (South), Manaus (North) and Campo Grande (Mid-west)	Each regional site recruited at least 12 primary caregivers. Participant recruitment occurred in April 2024	Schools of rural and urban area were defined by the education secretary of each city that participated. In general, urban and rural area was defined by Brazilian Institute of Geography and Statistics (IBGE)	In-person interviews and telephone-based interviews	Nonrandomized. In-person interview first, telephone-based interview second
**Canada**				
Sites across Canada represented Statistics Canada’s national surveillance regions: Atlantic, Quebec, Ontario, Prairies and British Columbia, with site leads located in Vancouver, Edmonton, London, Ottawa, Montreal, Halifax and Wolfville	The target sample size was 96 primary caregivers (16 primary caregivers across 6 Canadian sites) to reach the target of 48 paired observations for each test modality. Recruitment was conducted from June 2023 to December 2023	Urban and rural classification was based on participants’ postal code and the corresponding census subdivision. Subdivisions with a population under 30 000 people were considered rural, aligning with rural and small community designations in Canada^13^	In-person interviews, telephone-based interviews, virtual video interviews, paper-based questionnaire and REDCap-based questionnaire	Participants were randomized to complete three out of five questionnaires. Four modality groups were created representing an exhaustive set of combinations testing: (i) the criterion measure (in-person interview); (ii) one interview modality (phone or virtual video) and (iii) one questionnaire modality (paper or REDCap). At the site level, covariate stratified technique was used to ensure each combination of sex and rurality was assigned to all four modality groups. Each participant was randomly assigned one of six possible orders of assessed modalities (e.g. criterion-interview-questionnaire; criterion-questionnaire-interview). At the site level, the R Shiny application was used for randomization
**India**				
Recruitment occurred at two sites: Vadu (rural) and Pune City (urban). Vadu was randomly selected from 10 rural villages which are a part of the Health and Demographic Surveillance system. In Pune City, two pre-schools were randomly selected from a list of 10 pre-schools	Each site (Vadu and Pune City) recruited 24 primary caregivers. Recruitment was conducted from January 2025 to February 2025	Urban and rural classification were based on participants’ postal code and as per the definition of Census of India. Urban areas are characterized by municipalities, corporations, cantonment boards or notified town area committees with a minimum population of 5 000 and population density of at least 400 people per square kilometre. Rural areas are generally defined as areas that do not meet the criteria for urban areas, including villages with clear boundaries but no municipal board characterized by a lower population density and more than 25% of the male working population is engaged in agriculture^13^	In-person interviews and telephone-based interviews	Participants randomly assigned to either in-person or telephone-based interview completed first, using covariate stratified technique to ensure equal proportions of every sex and rurality combination completing each modality. Randomization occurred using the R shiny randomization package modified to accommodate the protocol in India
**Malawi**				
Data were collected from both rural and urban sites within Zomba district in southern Malawi	From each site, a total of 25 primary caregivers were recruited. Recruitment took place between October 2023 and November 2023	Urban and rural settings were classified according to definitions established by the Malawi National Statistical Office^14^	In-person interviews and telephone-based interviews	Non-randomized. Urban participants completed telephone interviews first before arranging an in-person interview, while rural participants completed an in-person interview first before the telephone interview. These two modalities were selected for use in Malawi due to limited access to smartphones, computers and the internet, which precluded virtual video interviews, while low literacy levels limited the feasibility of self-directed administration^15^^,^^16^

Note: Recruitment and data collection for all countries occurred from June 2023 to February 2025. All countries examined the criterion in-person interview and had a 1-week washout period between each tested modality.

Although we allowed country-level adaptations to the protocol, we kept some key components fixed. To align with the SUNRISE study’s sex- and rurality-balanced sampling strategy, we required the teams in all countries to recruit equal numbers of participants by sex and urban or rural status. We also allowed countries to drop modalities that were not feasible, such as online questionnaires in areas with limited internet access, but we required the criterion in-person interviews to be conducted in all countries.

The target sample size was 48 paired observations per country for each test modality to achieve 80% power for calculating mean differences, assuming an expected correlation of 0.8 and an intraclass correlation coefficient above 0.7.^18^^,^^19^ Table 1 provides further details on country-specific study design elements.

### Analyses

We examined whether overall ECDI2030 scores were equivalent across the in-person interview criterion assessment and the other tested modalities by calculating paired *t*-tests and intraclass correlation coefficients using a two-way mixed effects, single rater, absolute agreement model. We repeated these analyses after stratifying by country, sex and urban or rural status. We assessed the effect size of differences, including in the stratified analyses, using Cohen’s *d*. In the Canadian data set, we tested multiple modalities; therefore, we also compared the interview-based modalities (telephone and virtual video) and self-directed modalities (paper and online primary caregiver questionnaires) with in-person interviews using paired *t*-tests and intraclass correlation coefficients. We classified ECDI2030 scores as developmentally on track when children aged 36–41 months scored ≥ 11/20, when children aged 42–47 months scored ≥ 13/20 and when children aged 48–60 months scored ≥ 15/20.^6^ We assessed discordance in developmentally on-track classifications between the criterion and tested modalities using McNemar’s test.

To examine if ECDI2030 responses were equivalent at the item-level when comparing the in-person interview criterion assessment with other tested modalities, we used McNemar’s test to examine discordance in dichotomous scores (questions 1–18: yes versus no; question 19: not daily versus daily; and question 20: not at all, the same or less versus more and a lot more) and don’t know versus responded to the question. We also used McNemar’s test to compare administration difficulties across the different interview modalities. We considered results for *t*-tests and McNemar’s tests significantly different at *P*-values: ≤ 0.05 and intraclass correlation coefficients significantly similar at *P*-values: ≤ 0.05. We conducted all analyses in R (R Foundation, Vienna, Austria).

## Results

**Fig. 1:**
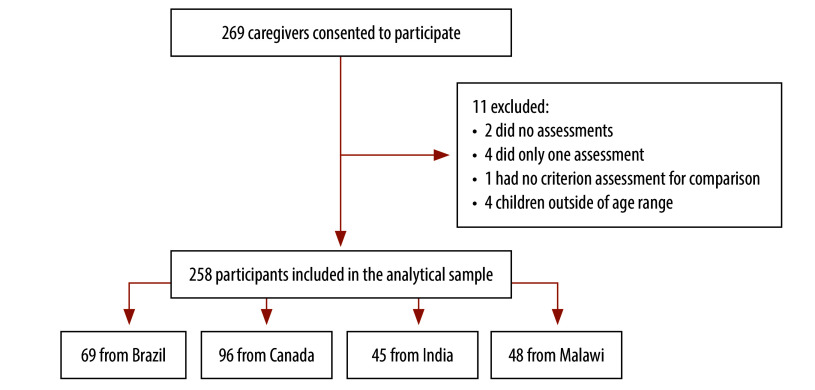
Flowchart on participant inclusion for the study on Early Childhood Development Index 2030 modalities, Brazil, Canada, India, Malawi, June 2023–February 2025

Note: 258 participants included in the analytical sample had at least two assessments, of which one is the in-person interview.

We obtained consent from 269 primary caregivers and included 258 individuals in the final analytical sample, with 212 paired observations from in-person and telephone interviews (Fig. 1 and Table 2). Across the four countries, the mean age of primary caregivers was 32 to 37 years and mothers accounted for 69% (33/48) to 91% (63/69) of caregivers. Children’s mean age ranged from 47 to 51 months (Table 2).

**Table 2:** Participant characteristics of the study on Early Childhood Development Index 2030 modalities, Brazil, Canada, India, Malawi, June 2023–February 2025

Characteristic	No. (%)^a^			
	Brazil (*n* = 69 children and 69 caregivers)	Canada (*n* = 96 children and 96 caregivers)	India (*n* = 45 children and 45 caregivers)	Malawi (*n* = 48 children and 48 caregivers)
**Age**				
Caregiver, years (SD)	33.7 (8.1)	36.5 (4.4)	32.0 (6.7)	34.6 (10.7)
Child, months (SD)	50.5 (6.6)	46.6 (7.0)	48.2 (7.6)	48.1 (7.4)
**Sex of child^b^**				
Female	36 (52.2)	47 (49.0)	22 (48.9)	24 (50.0)
Male	33 (47.8)	49 (51.0)	23 (51.1)	24 (50.0)
**Ethnicity^c^**				
Black	6 (8.7)	1 (1.0)	0 (0.0)	48 (100.0)^d^
Brown	32 (46.4)	NA	NA	NA
East Asian	NA	3 (3.1)	0 (0.0)	NA
Latin American	NA	1 (1.0)	0 (0.0)	NA
Middle Eastern	NA	3 (3.1)	0 (0.0)	NA
Multiple	NA	10 (10.4)	0 (0.0)	NA
South Asian	NA	1 (1.0)	45 (100.0)	NA
South-East Asian	NA	0 (0.0)	0 (0.0)	NA
White	31 (44.9)	77 (80.2)	0 (0.0)	NA
**Residence**				
Rural	36 (52.2)	46 (47.9)	24 (53.3)	24 (50.0)
Urban	33 (47.8)	50 (52.1)	21 (46.7)	24 (50.0)
**Caregiver relation**				
Mother	63 (91.3)	83 (86.5)	34 (75.6)	33 (68.8)
Father	4 (5.8)	13 (13.5)	9 (20.0)	10 (20.8)
Grandmother	2 (2.9)	0 (0.0)	1 (2.2)	4 (8.3)
Grandfather	0 (0.0)	0 (0.0)	1 (2.2)	0 (0.0)
Legal guardian	0 (0.0)	0 (0.0)	0 (0.0)	1 (2.1)
**Highest level of household caregiver education**				
No formal schooling	0 (0.0)	0 (0.0)	0 (0.0)	2 (4.2)
Primary or elementary	9 (13.0)	0 (0.0)	0 (0.0)	19 (39.6)
Secondary or high school	43 (62.3)	1 (1.0)	19 (42.2)	18 (37.5)
Vocational/college	1 (1.4)	16 (16.7)	9 (20.0)	0 (0.0)
Tertiary/university	16 (23.2)	79 (82.3)	16 (35.6)	9 (18.8)
Missing	0 (0.0)	0 (0.0)	1 (2.2)	0 (0.0)

NA: not applicable.

^a^ No. (%) presented if not otherwise stated.

^b^ As reported by caregiver.

^c^ The assessed ethnicity categories varied by country.

^d^ The Malawi team used a derived variable (black) based on survey options for Chewa, Tumbuka, Lomwe, Tonga, Yao, Sena, Ngonde, Ngoni and other.

While Canada examined all five modalities, Brazil, India and Malawi examined only in-person and telephone interviews due to concerns about literacy and internet access. Therefore, the main comparisons focus on these two modalities. Table 3 presents in-person and telephone ECDI2030 scores by country and site, showing strong agreement in each country and site. 

**Table 3:** Early Childhood Development Index 2030 scores by modality, Brazil, Canada, India, Malawi, June 2023–February 2025

Site	No. of interviews	Score^a^ (SD)	
		In-person	Telephone
**Brazil**	69	15.4 (2.6)	15.5 (2.7)
Midwest	10	16.0 (1.9)	16.0 (1.8)
North-east	16	15.4 (2.9)	15.6 (2.9)
North	20	15.6 (2.6)	15.7 (2.8)
South-east	11	15.5 (2.8)	15.3 (3.1)
South	12	14.8 (2.6)	14.8 (2.7)
**Canada**	96	17.2 (2.0)	17.0 (2.0)
British Columbia	15	17.7 (1.6)	17.1 (1.6)
Alberta	17	17.2 (2.2)	17.4 (2.3)
London, Ontario	16	16.8 (2.2)	16.9 (2.0)
Ottawa, Ontario	16	17.6 (2.0)	17.3 (2.0)
Quebec	15	16.5 (2.3)	16.3 (1.8)
Atlantic	17	17.4 (1.7)	16.9 (2.4)
**India**	45	16.6 (2.5)	16.1 (2.4)
**Malawi**	48	14.0 (3.4)	13.8 (3.6)
**Total**	**258**	**16.0 (2.8)**	**15.6 (2.9)**

SD: standard deviation.

^a^ The maximum score is 20.

Pooled data from all countries showed no significant differences in overall scores between in-person and telephone interviews as assessed by paired *t*-tests (Table 4). However, in stratified analyses, we found significantly higher scores from in-person interviews than from telephone interviews for female children (mean difference: 0.23, Cohen’s *d*_diff_: 0.22) and participants from India (mean difference: 0.47, Cohen’s *d*_diff_: 0.44). In contrast, we found lower scores among Canadian participants on the second completed assessment (mean difference: −0.27, Cohen’s *d*_diff_: −0.36), regardless of modality. All significant differences had small effect sizes.^20^ Pooled and stratified intraclass correlation coefficient results nonetheless indicated strong agreement between the two modalities, with values ranging from 0.89 to 0.93, which we considered good to excellent.^21^ Based on ECDI2030 scores, more than 80% of all children were classified as developmentally on track. Further, we found no significant differences in developmental on-track classifications between in-person and telephone interviews in either pooled or stratified comparisons.

**Table 4:** Comparing in-person and telephone interview Early Childhood Development Index 2030 scores, Brazil, Canada, India, Malawi, June 2023–February 2025

Variable	No. of comparisons^a^		Score by type of interview					Intraclass correlation coefficient^b^ (95% CI)		Developmentally on-track, No. (%)^c^	
			Criterion (in-person)	Comparison (telephone)	Mean ∆	*P*					
										In-person	Telephone
**All**	212		15.708	15.608	0.10	0.186		0.93 (0.91 to 0.95)		177 (83.49)	174 (82.08)
**Sex**											
Female	106		15.840	15.613	0.23	0.026		0.93 (0.89 to 0.95)		91 (85.85)	86 (81.13)
Male	106		15.575	15.604	−0.03	0.796		0.93 (0.90 to 0.95)		86 (81.13)	88 (83.02)
**Rurality**											
Rural	106		14.934	14.708	0.23	0.061		0.92 (0.89 to 0.95)		79 (74.53)	76 (71.70)
Urban	106		16.481	16.509	−0.03	0.749		0.93 (0.89 to 0.95)		98 (92.45)	98 (92.45)
**Country**											
Brazil	69		15.435	15.507	−0.07	0.582		0.91 (0.86 to 0.95)		53 (76.81)	54 (78.26)
Canada	50		16.940	17.020	−0.08	0.471		0.93 (0.87 to 0.96)		50 (100.00)	50 (100.00)
India	45		16.600	16.133	0.47	0.005		0.89 (0.79 to 0.94)		43 (95.56)	41 (91.11)
Malawi	48		13.979	13.792	0.19	0.322		0.93 (0.88 to 0.96)		31 (64.58)	29 (60.42)
**Order of comparison^d^**											
All	258		15.946	15.996	−0.05	0.439		0.93 (0.91 to 0.95)		222 (86.05)	221 (85.66)
Brazil	69		15.435	15.507	−0.07	0.582		0.91 (0.86 to 0.95)		53 (76.81)	54 (78.26)
Canada	96		17.052	17.323	−0.27	0.001		0.92 (0.87 to 0.95)		96 (100.00)	96 (100.00)
India	45		16.400	16.333	0.07	0.701		0.89 (0.81 to 0.94)		42 (93.33)	42 (93.33)
Malawi	48		14.042	13.729	0.31	0.096		0.93 (0.88 to 0.96)		31 (64.58)	29 (60.42)

^a^ Number of comparisons made between in-person (criterion) and telephone interviews (comparison), except for order of comparison.

^b^ Intraclass correlation coefficients show how closely individual ratings from the same raters agree, considering differences between subjects and raters and requiring exact score agreement. The specific intraclass correlation coefficients calculated for this study were two-way mixed effects, single rater, absolute agreement (random and mixed are equivalent but differ when interpreting).

^c^ No significant differences found when comparing developmentally on-track classifications, according to McNemar’s test.

^d^ The order of comparison was the first and second assessments completed, while treating the first order as the criterion. For instance, if a site was randomized to conduct in-person interviews first then this would be the first order or criterion, while if another site was randomized to conduct home-based interviews first then this would be that site’s first order condition or criterion that was compared to the second assessed modality.

In Canada, across all five modalities we examined, we found scores ranging from 16.14 to 18.43 out of 20 (online repository).^22^ Consistent with the Canadian comparison of first and second order assessments, we found that scores for the third assessments were significantly lower than those for the first (mean difference: −0.27, Cohen’s *d*_diff_: −0.35), regardless of modality (online repository).^22^ When comparing assessments by type (i.e. administered and self-directed) and specific modality, we found no significant differences, relative to in-person interviews (online repository).^22^ Intraclass correlation coefficient tests also showed that comparisons by order, type and specific modality were statistically similar.

McNemar’s tests showed no significant differences across the various modalities in any countries when comparing binary scores (e.g. yes and no) for individual questions in the ECDI2030 (online repository).^22^ These tests also showed no significant differences across all modalities when comparing don’t know responses with any other response category (online repository).^22^

Only Canada and India completed the administration difficulties questionnaire for the interview modalities (online repository).^22^ In India, 0% (0/45) to 2% (1/45) of responses indicated an administration difficulty compared with 0% (0/50) to 27% (13/49) for Canada. Specifically, Canadian parents were more likely to report interruptions or disruptions during in-person interviews (26.5%; 13/49) than during telephone interviews (4.1%; 2/49; *P*-value: 0.01). McNemar’s test detected no significant differences in administrative difficulties across the modalities (online repository).^22^

## Discussion

Across four diverse countries, the ECDI2030 produced equivalent results whether administered by telephone or in person. When we stratified the results by country, sex and rurality, small differences emerged in overall development scores. Across all sites, caregivers gave higher scores to females in in-person assessments than telephone assessments and caregivers from India gave higher scores in in-person assessments regardless of child sex. Canadian caregivers gave lower scores on the second assessment, regardless of modality. However, these statistically significant results had small effect sizes: the largest difference was only half a point on the 20-point scale. As such, these differences are unlikely to be meaningful in practice. Comparisons also remained statistically similar, as indicated by intraclass correlation coefficients and classifications of developmental on-track status. Indeed, all pooled and stratified assessments demonstrated statistically similar intraclass correlation coefficients, ranging from good to excellent, and similar frequencies of children classified as developmentally on track. The agreement of telephone and in-person ECDI2030 scores in Brazil, Canada, India and Malawi aligns with findings from a similar study in Nepal.^9^ In that study, researchers randomized caregivers to either telephone or in-person ECDI2030 interviews and found equivalent mean scores and developmental on-track classifications. Together, our findings strengthen the emerging evidence base that the ECDI2030 can be administered reliably through either in-person or telephone interviews.

The Canadian researchers developed the data collection protocol first. Rather than impose Canadian-centric aspects on collaborating countries, we preserved country-level autonomy to ensure a feasible method and useful findings. As a result, several important distinctions emerged. In Brazil and Malawi, the distance between rural sites and the locations where data collectors live posed a major logistical barrier. Therefore, to improve data collection feasibility, the team did not randomize modality order and instead implemented predetermined orders. Importantly, the analyses found no differences related to modality order in Brazil or Malawi. In addition, only the Canadian team examined several modalities, whereas the teams in Brazil, India and Malawi chose to compare only in-person interviews to telephone interviews. The investigators determined that other modalities would likely encounter barriers related to literacy (for self-directed questionnaires) and internet connectivity (for virtual video interviews), particularly in rural sites.

Although we conducted modality comparisons only in Canada, the findings may still be relevant to similar high-income countries. In Canada, both interviewer-administered modalities and self-directed questionnaires agreed with the criterion in-person interviews. Similarly, a study compared direct assessments and caregiver reports of development in 510 urban and rural preschool children (aged 36–59 months) across Bangladesh, China, India and Myanmar.^23^ Fifteen questions overlapped between the direct assessment and self-directed measures and the 20-item ECDI2030. Based on expected literacy rates, researchers in Bangladesh, India and Myanmar used interviews to collect caregiver reports, while in China, they used paper-based questionnaires. Among the caregivers of 156 Chinese preschoolers, the paper-based questionnaire showed a moderate, statistically significant correlation (*r*: 0.33, *P*-value: < 0.001) with direct observations.^23^ These findings suggest that, in countries where literacy is not perceived as a barrier, self-directed questionnaires could enhance the feasibility of data collection. This approach may be particularly useful in rural research settings, where the self-directed questionnaires could allow researchers to measure overall development across distances that would otherwise be difficult to manage. However, additional research is needed to determine whether these results generalize beyond Canada and China.

We found no significant item-level differences in binary scores or in the proportion of don’t know responses between in-person and telephone interviews. However, these results should be interpreted with caution because the current sample lacked sufficient power to rule out true item-level differences with confidence. Despite this limitation, we showed that the ECDI2030 could be feasibly implemented within the SUNRISE data collection protocol.^11^ In planned work, we will measure development with the ECDI2030 alongside more direct indicators, such as gross motor skills in 4000 children from urban and rural settings across Brazil, Canada, India and Malawi. Future SUNRISE analyses will not only compare item-level responses across self-selected modalities but will also enable sufficiently powered investigations of developmental measures and key demographic contrasts, such as caregiver education.

A main strength of this study was the collection of data from four economically, geographically and culturally diverse countries and inclusion of multiple sites within each country to improve representativeness, while balancing recruitment by sex and urban or rural status. Another strength was that in two of the four countries we randomized modality order; in the two countries where we did not, we found equivalent overall development scores and on-track classifications when comparing first and second assessment orders. A major limitation of this study was that the age range (36–60 months) did not include toddler-aged children (24–35 months), for whom the ECDI2030 is also validated. As a result, it remains unclear whether agreement across modalities extends to younger children. Although we recruited balanced samples across child sex and urban or rural residence from multiple sites, important differences from the general populations may still exist in each country. For example, the proportion of households with at least one caregiver who had attained tertiary education exceeded national averages for individual attainment, according to calculations from national surveys (Brazil 24.6% versus 22.7% year 2024; Canada 99.0% versus 49.3% year 2021; India 55.6% versus 12.4% year 2021; and Malawi 18.4% versus 2.0% year 2020).^24^

In conclusion, this multinational, multicentre, crossover study demonstrates strong agreement between telephone and in-person administration of the ECDI2030 across economically, culturally and geographically diverse countries. However, more research is needed to test whether these findings apply to younger children and households with lower caregiver education. Canadian data also support agreement across other ECDI2030 modalities, including virtual video interviews and self-directed questionnaires, that may be more appropriate in settings where literacy and internet connectivity are not considered barriers. Broadening modalities for ECDI2030 administration would greatly improve feasibility in studies with geographical differences between rural areas and the central research site. Remote options (e.g. telephone interviews, virtual video interviews) could likewise enhance feasibility and recruitment in urban areas where caregivers often have limited time, and data collectors typically communicate with caregivers through childcare educators or electronic communication. By moving beyond in-person only data collection, researchers can redirect time and cost savings towards improving the reach and representativeness of data collection.
